# Lymphocytic Esophagitis Associated With Chronic Severe Psoriasis: A Case Report With Special Reference to Programmed Death-Ligand 1 Expression

**DOI:** 10.7759/cureus.70815

**Published:** 2024-10-04

**Authors:** Maria Koleva, Martin Raby, Dorian Dikov

**Affiliations:** 1 Department of General and Clinical Pathology, University Hospital St. George, Plovdiv, BGR; 2 Department of Pathology, Jossigny Hospital, Jossigny, FRA

**Keywords:** esophagus, immunology, lymphocytic esophagitis, programmed cell death-ligand 1, psoriasis

## Abstract

Lymphocytic esophagitis (LE) is a rare type of chronic esophagitis, marked by an increased number of peripapillary intraepithelial lymphocytes (IEL) with little to no presence of intraepithelial granulocytes and intercellular edema. There is currently no established standard for quantifying IEL in the esophageal mucosa.

Clinically, LE manifests with a range of symptoms, including difficulty swallowing (dysphagia), indigestion (dyspepsia), nausea, and chest pain. It is more commonly diagnosed in older women and is often associated with tobacco use, gastroesophageal reflux disease, and achalasia, although the exact cause remains unknown.

Programmed cell death protein 1 (PD-1) and its ligand (PD-L1) are expressed on the surface of immune and epithelial cells in healthy and tumor tissues, typically in response to various cytokines.

We present a new case of LE associated with chronic severe psoriasis, highlighting the role of PD-L1 expression. This supports the immunopathological basis of the disease and its links to other immune and autoimmune disorders, suggesting its potential as a biomarker for distinguishing LE from reflux esophagitis.

## Introduction

First described by Rubio et al. in 2006, lymphocytic esophagitis (LE) is a rare form of chronic esophagitis [1]. It has a distinct histologic phenotype characterized by an increased number of peripapillary intraepithelial lymphocytes (IEL) in the absence or minimal presence of intraepithelial granulocytes [2]. As a result, multiple studies on LE have observed intercellular edema, known as spongiosis, on pathology slides, leading to its inclusion in Rubio et al.’s definition. There is no consensus norm in the quantitative study of intra-epithelial esophageal mucosal lymphocytes. Some authors suggest a definition of LE included an increase of peripapillary IEL to more than 20 IEL per high power field, with minimal or no polymorphonuclear neutrophil granulocytes (PN), along with the presence of spongiosis [3].

The clinical presentation of LE varies and can include symptoms such as dysphagia, dyspepsia, nausea, or chest pain. It is often diagnosed in older women and is associated with a history of tobacco use, gastroesophageal reflux disease (GERD), and achalasia [2].

We describe a new case of LE with special reference to programmed death-ligand 1 receptor (PD-L1) expression. Based on the presumption that similar to other chronic inflammatory diseases of immune and autoinflammatory pathogenesis [4,5], the expression of PD-L1 will be enhanced and could serve as an additional diagnostic and differential diagnostic aid. This is the first attempt to examine PD-L1 expression in LE.

## Case presentation

A 40-year-old female patient presented with GERD, exhibiting both typical symptoms (heartburn and acid regurgitation) and atypical symptoms (chronic cough and non-cardiac chest pain), including halitosis. The patient's medical history is significant for psoriasis, diagnosed at the age of 14, previously managed with dermocorticoids, Daivobet, phototherapy, and methotrexate, with treatment discontinued after partial improvement. Psoriasis has worsened over the past year, and the patient is currently using Enstilar® and Diprosone® lotion for management. The patient has no evidence of cutaneous or mucosal lichen planus.

**Figure 1:**
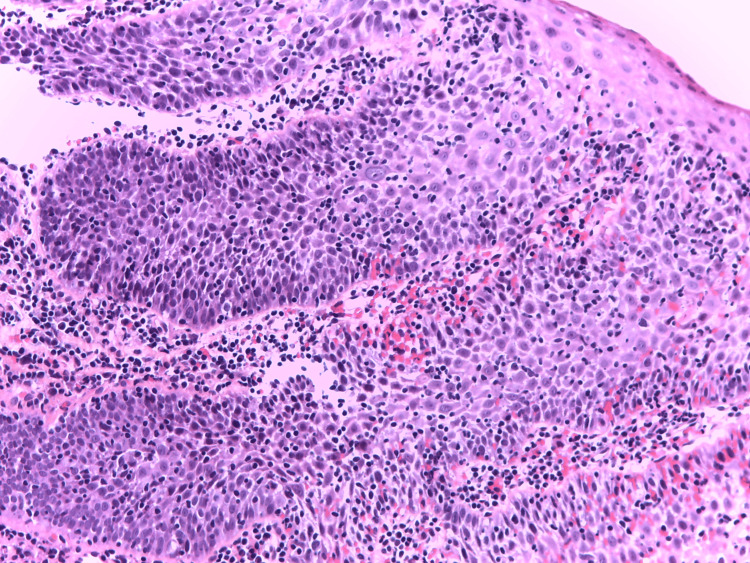
Non-keratinizing squamous epithelium exhibiting marked spongiosis, accompanied by an inflammatory infiltrate primarily consisting of lymphocytes: hematoxylin-eosin-saffron, x200.

**Figure 2:**
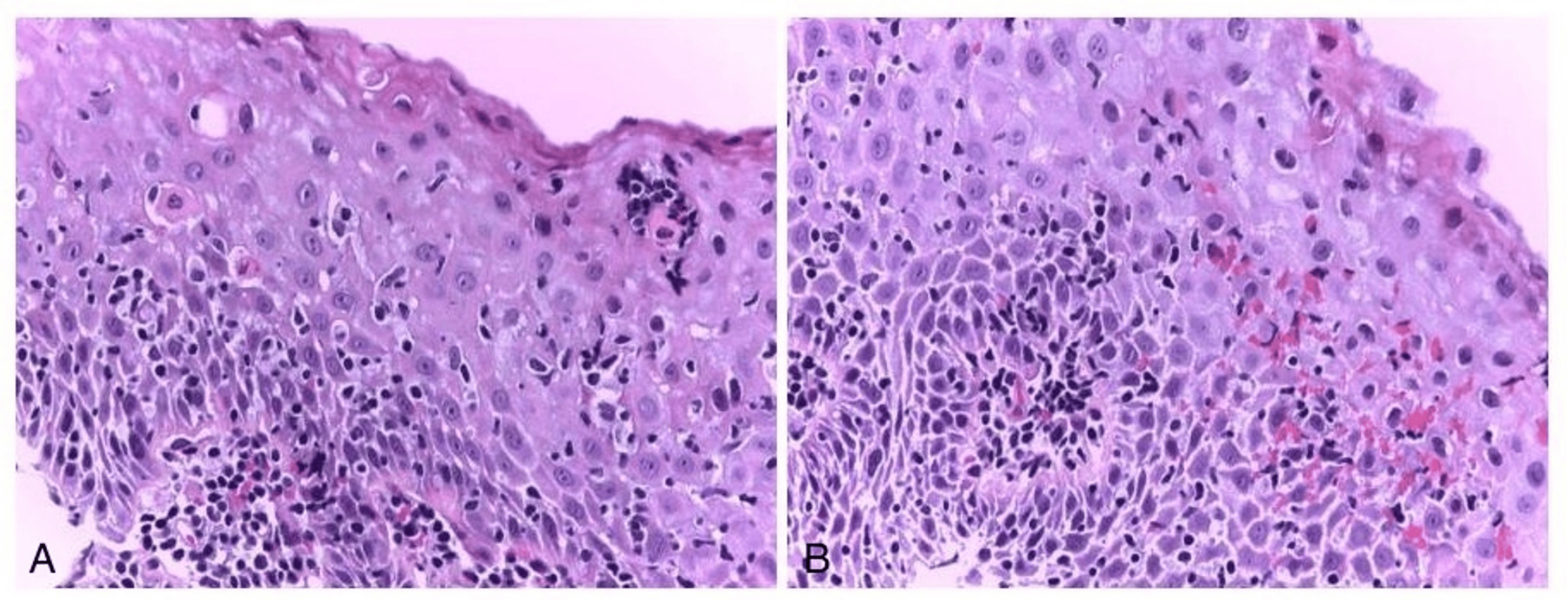
Histological findings reveal apoptotic keratinocytes (A) accompanied by a small number of polymorphonuclear neutrophil granulocytes (B). A: hematoxylin-eosin-saffron, x400; B: hematoxylin-eosin-saffron, x400

**Figure 3:**
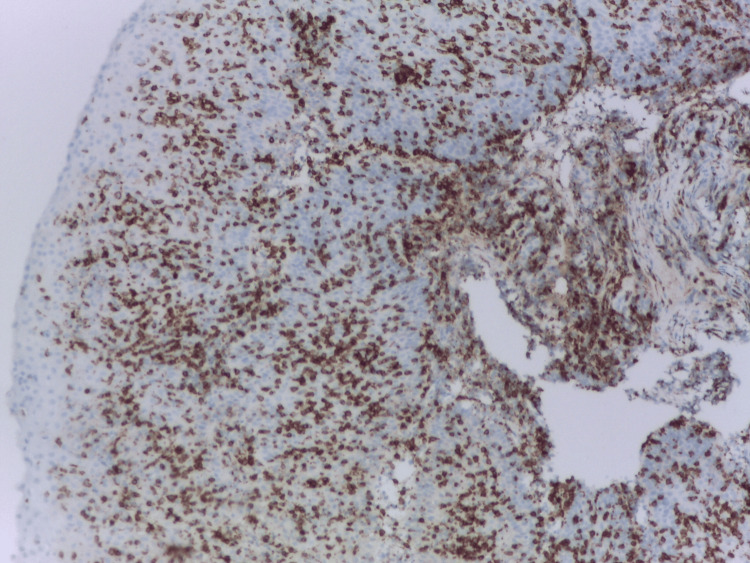
Positive CD8+ expression in intraepithelial lymphocytes. Immunohistochemistry anti-CD8, x200.

**Figure 4:**
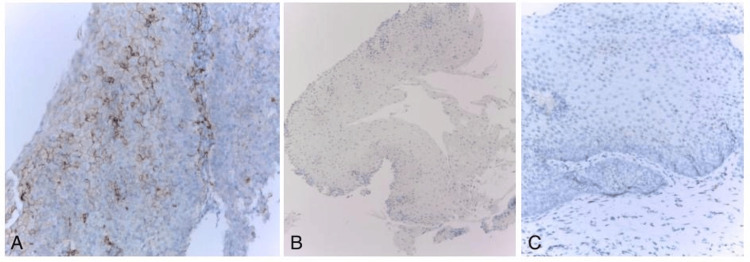
PD-L1 expression in LE and in control groups (normal esophageal mucosa and reflux esophagitis): strong membranous PD-L1 expression in the foci of increased intraepithelial lymphocytic inflammatory infiltrates (A). Lack of PD-L1 immunoreactivity in the control groups: normal mucosa (B) and reflux esophagitis (C). Immunohistochemistry anti-PD-L1. A: x200; B: x40; C: x200

Esophagogastroduodenoscopy (EGD) revealed normal duodenal mucosa and moderate inflammatory antral and fundic changes confirmed by biopsies, initially positive for *Helicobacter pylori* (HP), but after eradication treatment, five months later, HP is no longer observed. EGD revealed also two small, millimetric areas of endobrachyesophagus (C-M1 Prague classification) in the lower third of the esophagus. A biopsy from this area was taken. Histopathological analysis demonstrated a non-keratinizing squamous epithelium with notable spongiosis and an inflammatory infiltrate predominantly composed of lymphocytes (Figure 1). Apoptotic keratinocytes were present, along with a small number of PN (Figures 2a, 2b). Additional findings included basal epithelial hyperplasia. There was no evidence of pathogenic microorganisms, viral cytopathic effects, ulceration, metaplasia, or dysplasia. An immunofluorescent investigation for the diagnosis of lichen was not performed. Immunohistochemistry revealed lymphocytes expressing CD3+ (Bond ready-to-use antibody, clone LN10, Leica Biosystems, Germany) (>40/1HPF), CD8+ (Bond TM ready-to-use antibody, clone 4b11, Leica Biosystems) (Figure 3), and CD4+ (1:100, clone SP35, Cell Marque, Rocklin, USA), with negative expression for CD20- (Bond ready-to-use antibody, clone LN26, Leica Biosystems) (data not shown). The histological and immunohistochemical findings are indicative of LE, though the presence of concurrent reflux-induced lesions could not be definitively excluded (Figure 2b). In this regard, we also include an additional examination of PD-L1 expression (1:200, clone QR1, BIOCYC, Potsdam, Deutschland). In our case, we found strong membranous PD-L1 expression in the areas of pronounced IEL inflammatory infiltrate (Figure 4a) (combined positive score (CPS) = 72). For the control group, we used six biopsy specimens from esophageal mucosa: three with intact mucosa, two with proven reflux esophagitis, and one with candida esophagitis. In the control groups of patients, it was not found significant expression of PD-L1 (CPS < 1) (Figures 4b, 4c).

A follow-up gastroscopy showed consistent histological findings in the esophagus.

## Discussion

Lymphocytes are typically found in the squamous mucosa of the esophagus. Most of these are CD3+CD8+ T cells with a suppressor cytotoxic phenotype. They are usually more abundant in the suprabasilar region of the epithelium and often appear convoluted or squiggle in shape [6].

LE is a rare form of chronic esophagitis with an unknown etiology. The basic morphological finding is increased IEL, although there can also be varying amounts of neutrophils, which is why there are no established criteria differentiating it from other types of esophagitis. It remains unclear whether LE is a distinct primary inflammatory disease of the esophagus or simply an indicator of an underlying esophageal or systemic immune-mediated disease process [7].

Clinically, the majority of patients diagnosed with histologically confirmed LE are older females or patients with underlying immunologic conditions such as Crohn's disease, rheumatologic disorders, or common variable immunodeficiency [8]. In the case presented by us, the patient is relatively young, which can likely be attributed to the long-standing medical history (26 years) of an immunocompromised condition - psoriasis.

Psoriasis is an immune-mediated or autoinflammatory disorder characterized by erythematous papules and plaques with pronounced scaling on the skin. Patients with psoriasis often experience numerous comorbidities, including psoriatic arthritis, inflammatory bowel disease, autoimmune ocular disorders, and, most significantly, metabolic syndrome with associated cardiovascular complications.

The pathogenesis of psoriasis is complex and remains partially understood despite extensive research. A key factor in psoriasis is chronic T-cell activation by antigen-presenting cells, though the exact mechanism is unclear. One theory links psoriasis to disruptions in immunotolerance, which is normally maintained through mechanisms like central negative selection of autoreactive T cells and peripheral suppression. The programmed cell death protein 1 (PD-1) co-receptor, found on immune cells, inhibits effector T cells and supports T-regulatory cells to maintain self-tolerance. PD-1 dysfunction may contribute to the chronic T-cell hyperactivation seen in psoriasis, though research on PD-1/PD-L1 involvement remains limited and inconsistent [9].

In our case, such PD-L1 investigation on psoriasis has not been done; therefore, we have decided to investigate PD-L1 expression in LE. The use of PD-L1 as a diagnostic marker does not need a quantitative study. We used the known tumoral pathology CPS for scoring the PD-L1 expression.

PD-L1 expression is induced at the surface of immune and epithelial cells of healthy and tumor tissues in response to various cytokines [10]. The role of PD-L1 in the dysregulation of T-helper immune responses observed in LE is poorly investigated and unclear. There were only several investigations concerning PD-L1 expression in gut mucosa. While significant progress has been made in the understanding of the role of PD-L1 in maintaining gut homeostasis and its involvement in inflammatory disease, the knowledge remains incomplete. It is well established that PD-L1 plays a critical role in gut mucosal tolerance. Abnormalities in PD-L1 expression and/or signaling have been documented in chronic inflammatory gut conditions such as Crohn’s disease, ulcerative colitis, and celiac disease, as well as in chronic infections such as HP-gastritis [4].

The findings of this study demonstrate a lack of PD-L1 expression in the normal and reflux esophagitis mucoses and a notable increase in PD-L1 expression in the case involving LE. The marked expression of PD-L1 in LE confirms its immune or autoinflammatory nature. The lack of PD-L1 expression in reflux esophagitis is probably a reflection of the chemical irritant pathogenesis of this disease. Apparently, PD-L1 expression is correlated with mucosal esophageal infiltration by PN and can be used for diagnosis of LE and differential diagnosis with reflux esophagitis.

## Conclusions

In conclusion, we report a new case of LE associated with chronic severe psoriasis with special reference to PD-L1 expression. This supports the immunopathological nature of the disease and its association with other immune and autoimmune disorders. Furthermore, it suggests that PD-L1 could serve as a potential marker for differential diagnosis in cases involving PN, distinguishing it from one of the most common other types of esophagitis - reflux esophagitis.
